# Ferroptosis Regulator Modification Patterns and Tumor Microenvironment Immune Infiltration Characterization in Hepatocellular Carcinoma

**DOI:** 10.3389/fmolb.2022.807502

**Published:** 2022-01-28

**Authors:** Dong-Li Liu, Ming-Yao Wu, Tie-Ning Zhang, Chun-Gang Wang

**Affiliations:** Department of Radiation Oncology, Shanghai General Hospital, Shanghai Jiao Tong University School of Medicine, Shanghai, China

**Keywords:** hepatocellular carcinoma, ferroptosis modification, tumor microenvironment, immune, prognosis

## Abstract

Accumulating studies have highlighted the biologic significances of ferroptosis modification in tumor progression, but little is known whether ferroptosis modification patterns have potential roles in tumor microenvironment (TME) immune cell infiltration of hepatocellular carcinoma (HCC). In this study, we evaluated 51 ferroptosis regulators and performed consensus clustering algorithm to determine ferroptosis modification patterns and the ferroptosis related gene signature in HCC. Gene set variation analysis (GSVA) was employed to explore biological molecular variations in distinct ferroptosis modification patterns. Single sample gene set enrichment analysis (ssGSEA) algorithm was performed to quantify the relative infiltration levels of various immune cell subsets. Principal component analysis (PCA) algorithm was used to construct the ferroptosisSig score to quantify ferroptosis modification patterns of individual tumors with immune responses. Three distinct ferroptosis modification patterns were identified. GSVA enrichment analysis indicated that three ferroptosis modification subgroups were enriched in different metabolic pathways. ssGSEA analysis determined that 19 of 24 immune infiltrating cells had significant differences in three distinct ferroptosis patterns. A 91-ferroptosis gene signature was constructed to stratify patients into two ferroptosisSig score groups. Patients in the higher ferroptosisSig score were characterized by significantly prolonged survival time compared with patients in the lower ferroptosisSig score group (*p* < .0001). An immunotherapy cohort confirmed patients with higher ferroptosisSig score determined significant therapeutic advantages and clinical benefits. Receiver operating characteristic (ROC) curve analysis confirmed the predictive capacity of anti-PD/L1 immunotherapy by ferroptosisSig score. Our study indicated the ferroptosis modification played a significant role in TME heterogeneity and complexity. Evaluating the ferroptosis modification pattern of individual tumor could strengthen our cognition of TME infiltration characteristics and guide more effective clinic immunotherapy strategies.

## Introduction

Ferroptosis is a novel discovered type of non-apoptotic-regulated programmed cell death induced by the iron-dependent accumulation of lipid reactive oxygen species (ROS) of metabolic dysfunctions (Dixon et al., 2012). Previous studies determined that multiple factors such as amino acids, lipids and iron metabolism could affect the initiation and execution of ferroptosis (Stockwell et al., 2017). The ferroptosis modifications are regulated by ferroptosis regulator genes (FRGs), including both suppressors of ferroptosis (SOFs) and drivers of ferroptosis (DOFs). Growing evidence indicated that dysregulated expression and genetic variations of FRGs were associated with the disorders of various biological process including tumor carcinogenesis, cell death and proliferation dysregulation, tumor progression and immunomodulatory abnormality (Yagoda et al., 2007; Yang et al., 2014; Sun et al., 2016; Hassannia et al., 2019). An in-depth understanding of different ferroptosis modifications and expression perturbations underlying cancer heterogeneity could further benefit the identification of clinical therapeutic targets.

Hepatocellular carcinoma (HCC) is estimated to be the sixth most prevalent cancer worldwide and the third leading cause of cancer-related death (Sung et al., 2021). Despite improvements in early detection and clinical management of HCC over the last decade, the highly lethal ratio is still a growing concern (Villanueva, 2019). In recent years, tumor immunotherapy based on immune checkpoint inhibitors (ICIs) has achieved considerable success clinical efficacy. Plenty of phase I and II clinical trials suggest that immune-based therapies, such as anti-PD-1/PD-L1/CTLA-4 monoclonal antibody strategies, could benefit the patients with HCC (Hilmi et al., 2019). Based on the encouraging results in unresectable HCC from a phase Ib study, the combination of lenvatinib plus pembrolizumab has been granted a breakthrough therapy designation by the United States Food and Drug Administration (Finn et al., 2020a). Nevertheless, the two phase III trials KEYNOTE-240 (pembrolizumab versus placebo in second line) (Finn et al., 2020b) and CheckMate 459 (nivolumab versus sorafenib in first line) (Yau et al., 2019), failed to reach their primary survival endpoints in advanced HCC. HCC cells could induce various biological behavior changes through direct and indirect complex interactions with tumor microenvironment (TME) including inhibiting apoptosis, inducing proliferation as well as immune evasion. Therefore, comprehensive analyses of TME landscape heterogeneity and complexity might be helpful to identify different tumor immune-phenotypes and improve the ability to guide and predict immunotherapy responsiveness.

Recently, many studies reported the interactions between TME infiltration immune cells and ferroptosis modifications. Wang et al. (2019) reported that immunotherapy activated CD8^+^ T cells enhance ferroptosis-specific lipid peroxidation in tumor cells by down regulated SLC7A11 and SLC3A2, and that increased ferroptosis contributes to the anti-tumor effect of immunotherapy. Some studies determined that anti-PD-L1 monoclonal antibodies and ferroptosis activators, such as erastin, RSL3 and cyst(e)inase, synergistically suppressed tumor growth *in vitro* and *in vivo* (Ghoochani et al., 2021). As a critical ferroptosis regulator, HMGB1 knockdown involved in tumor immunity through the RAS-JNK/p38 pathway (Ye et al., 2019). In addition, HMGB1 released from ferroptotic tumor cells could trigger M1 polarization by activating the HMGB1-AGER pathway (Ye et al., 2019). However, the TME cell infiltration characteristics mediated by ferroptosis regulators in HCC is still not well understood and further studies are essential to unveil the underlying mechanisms.

In the present study, we evaluated the association between ferroptosis modification patterns and TME immune cell infiltration characteristics of HCC samples from public databases. Moreover, we constructed a developed a scoring scheme to quantify the ferroptosis modification pattern of individual HCC patients. These findings suggest that the ferroptosis modification plays a vital role in TME profiles and in directing more effective treatment strategies for HCC.

## Materials and Methods

### Hepatocellular Carcinoma Dataset Source and Preprocessing

The workflow of our study was shown in Supplementary Figure S1A **.** Gene expression data and clinical features of HCC samples were retrospectively searched in Gene Expression Omnibus (GEO) database (https://www.ncbi.nlm.nih.gov/geo/), The Cancer Genome Atlas (TCGA) database (https://cancergenome.nih.gov/) and International Cancer Genome Consortium (ICGC) database (https://icgc.org/). Datasets including single sample more than 150 patients and containing complete survival information were considered in this study. In total, three eligible HCC cohorts [GSE76427 (N = 167), LIRI-JP (N = 445) and TCGA-LIHC (The Cancer Genome Atlas- Liver Hepatocellular Carcinoma, N = 427)] were collected in this study for further analysis (Supplementary Table S1). For microarray data from Affymetrix platform, we downloaded the raw “CEL” files and carried out background adjustment and quantile normalization with the affy and simpleaffy packages. For microarray data from other platforms, we directly downloaded the normalized matrix files. As to data from TCGA, we using the R package TCGAbiolinks to download RNA sequencing data (FPKM format) of gene expression from the Genomic Data Commons (GDC, https://portal.gdc.cancer.gov/) (Colaprico et al., 2016). The “ComBat” algorithm of sva package were used to correct batch effects from non-biological technical biases. The clinical information of three cohorts are listed in Supplementary Table S2.

### Unsupervised Clustering for 51 Ferroptosis Regulators

A total of 51 regulators were extracted from TCGA、GEO and ICGC for identifying different ferroptosis modification patterns mediated by ferroptosis regulators. Unsupervised clustering analysis was used to identify distinct ferroptosis modification patterns based on the expression of 51 ferroptosis regulators and classify patients for further analysis. The number of clusters and their stability were determined by the consensus clustering algorithm (Hartigan et al., 1979). The R package of ConsensusClusterPlus was utilized to analyze the consensus clustering (Wilkerson and Hayes, 2010). We used “survminer” package to perform the KM survival analysis in distinct ferroptosis cluster patients.

### Gene Set Variation Analysis and Gene Ontology Annotation

We performed GSVA enrichment analysis using the R package of GSVA to explore the variation in pathway and biological processes in distinct ferroptosis modification patterns (Hänzelmann et al., 2013). The well-defined gene symbols were derived from MSigDB database for running GSVA analysis. Adjusted *p* < 0.05 was considered as statistically significance. We used clusterProfiler R package to perform functional annotation for ferroptosis phenotype-related genes with the cutoff value of FDR <0.05.

### Estimation of Immune Cell Infiltration

Single sample gene set enrichment analysis (ssGSEA) algorithm was performed to quantify the relative abundance of 24 immune infiltration cells in the tumor microenvironment of HCC. The R package of GSEABase was used to determine the infiltrating variation and prognosis in distinct ferroptosis modification patterns.

### Identification of Differentially Expressed Genes Between Distinct Ferroptosis Phenotypes

We classified HCC patients into three distinct ferroptosis modification patterns based on the expression of 51 ferroptosis regulatory genes by identifying ferroptosis related genes. The limma R package was used to evaluate DEGs in HCC samples between different modification subtypes (Ritchie et al., 2015). The significance criteria for determining DEGs was set as adjusted *p* value <0.05.

### Construction of the Ferroptosis Regulator Signature

We developed a set of scoring system to evaluate the ferroptosis modification pattern of individual patients with HCC and we termed it as ferroptosisscore. We utilized the R package of “randomForset”, “survminer” and “pca3d” to identify DEGs of ferroptosis gene signatures from different ferroptosis clusters. Next, we used univariate Cox regression model to analyze the prognostic analysis for each gene in the signature. We then performed principal component analysis (PCA) to generate ferroptosis releated gene signature. We extracted principal component 1 and 2 as the ferroptosis signature score. We then defined the ferroptosis score using a formula similar to previous studies (Zhang et al., 2020): ferroptosis score *= ∑*(*PC1*_*i*_ *+ PC2*_*i*_), where i is the expression of ferroptosis phenotype-related genes.

### Collection of Gene Expression Data With Immunotherapy

Immunophenoscore (IPS) is a superior predictor of response to anti-PD-L1 treatments, which could quantify the determinants of tumor immunogenicity and characterize the intratumoral (Charoentong et al., 2017). Two immunotherapeutic cohorts were finally included in our study: bladder cancer (IMvigor210 cohort) and hepatocellular carcinoma with intervention of anti-PD-L1 antibody (GSE14520 cohort downloaded from GEO). Bladder cancer cohort was employed to verify our ferroptosis score system and the complete expression data and clinical information was obtained from http://research-pub.Gene.com/imvigor210corebiologies. The raw data were normalized by the DEseq2 R package and transformed into the TPM value for further analysis.

### Statistical Analyses

Spearman and distance correlation analyses were used to compute correlations coefficients in immune infiltration cells of ferroptosis regulators. Kruskal-Wallis and one-way ANOVA analyses were used as nonparametric and parametric methods more than two groups (Hazra and Gogtay, 2016), respectively. Student’s t test was employed to analyze statistical significance for quantitative data. Wilcoxon *t* test was performed to compare two groups. The Kaplan-Meier and log-rank test were used to determine the significance of prognostic differences. The R package “RCircos” was applied to plot the copy number variation landscape of 51 ferroptosis regulators in 23 pairs of chromosomes (Mayakonda et al., 2018). The R package “Survminer” was employed to analyze the ferroptosis modification pattern and prognosis for the Cox proportional hazards model. The R package “corrplot” was applied to analyze the association between ferroptosis score and the enrichment pathway. The surv-cutpoint function of the “survival” R package was utilized to stratify samples into low and high ferroptosis score subgroups. The forestplot R package was used to visualize prognosis of 24 immune infiltrating cells in three distinct ferroptotsis modification cluster. The waterfall function from maftools package was adopted to present the landscape in patients of distinct ferroptosis subtype in TCGA-LIHC cohort. The receiver operating characteristic (ROC) curve was applied to evaluate the prognosis classification performance of the ferroptosis score model. The R package of pROC was employed to quantify the area under the curve (AUC). Software R (version 3.6.1) and R Bioconductor packages were utilized in this study. All statistical *p* value were two side and *p* < 0.05 was statistically significance.

## Results

### Landscape of Genetic Variation of Ferroptosis Regulators in Hepatocellular Carcinoma

**FIGURE 1:**
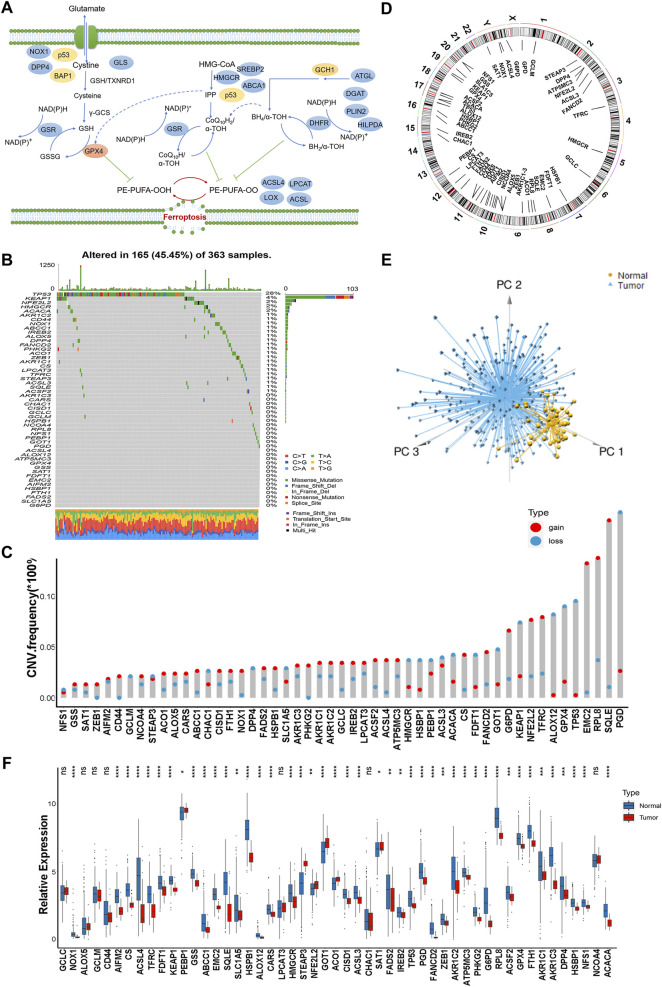
Landscape of genetic variation of ferroptosis regulators in hepatocellular carcinoma. **(A)** Summary of the dynamic process of ferroptosis mediated by regulators. **(B)** A total of 165 of 363 HCC samples experienced genetic alterations of ferroptosis regulators from TCGA-LIHC cohort. Each column represented individual patients. The number on the right showed the mutation frequency of each regulator. The barplot on the right indicated the proportion of each variant type. **(C)** The CNV variation frequency of ferroptosis regulators. The height of the column indicated the alteration frequency. The amplification frequency, red dot. The deletion frequency, blue dot. **(D)** The location of CNV alteration of ferroptosis regulators on chromosomes. **(E)** Principal component analysis of 51 ferroptosis regulator genes to distinguish tumors from normal samples. **(F)** The difference of mRNA expression levels of 51 ferroptosis regulators between normal and HCC samples. The asterisks represented the statistical *p*-value (*p < 0.05; **p < 0.01; ***p < 0.001).

In this study, we identified the roles of 51 ferroptosis regulatory genes in HCC. Figure 1A summarized the dynamic process of ferroptosis mediated by regulators (Figure 1A). We first investigated the incidence of copy number variations and somatic mutations of 51 ferroptosis regulator genes in HCC. A total of 165 of 363 (45.45%) samples experienced genetic alterations of ferroptosis regulators, primarily including missense mutations and deep deletions. TP53 exhibited the highest mutation frequency, followed by KEAP1 and NFE2L2 in HCC samples (Figure 1B). Next, the analysis of copy number variation (CNV) alteration frequency indicated that the CNV mutations were prevalent. SQLE, RPLB and EMC2 showed a widespread frequency of CNV amplification, while PGD, TP53 and GPX4 had prevalent CNV deletions (Figure 1C). The locations of CNV alterations of 51 ferroptosis regulator genes on chromosomes are shown in Figure 1D. Furthermore, we performed principal component analysis (PCA) based on the expression of 51 ferroptosis regulator genes and we could completely distinguished HCC samples from normal tissues (Figure 1E). Next, we found that 44 of 51 ferroptosis regulators were significantly differential expressed in HCC tissues compared with normal liver tissues in TCGA dataset. Further analysis showed that most of ferroptosis regulators such as ACSL4, EMC2, HSPB1, G6PD, RPLB, and TFRC were significantly decreased in HCC samples compared to normal specimens, while only five ferroptosis regulators including PBEB1, STEAP3, NFE2L2, GOT1, and AOC1 were markedly increased in HCC patients indicating that the expression of ferroptosis regulators with CNV alterations were the principal factors leading to disordered expressions of ferroptosis regulators (Figures 1C,F). Taken together, these findings demonstrated the significant heterogeneity in the genomic and transcriptomic landscape of ferroptosis regulators between normal and HCC samples suggesting a potential role for ferroptosis regulators in HCC genesis and progression.

### Identification of Ferroptosis Modification Patterns Mediated by 51 Regulators

**FIGURE 2:**
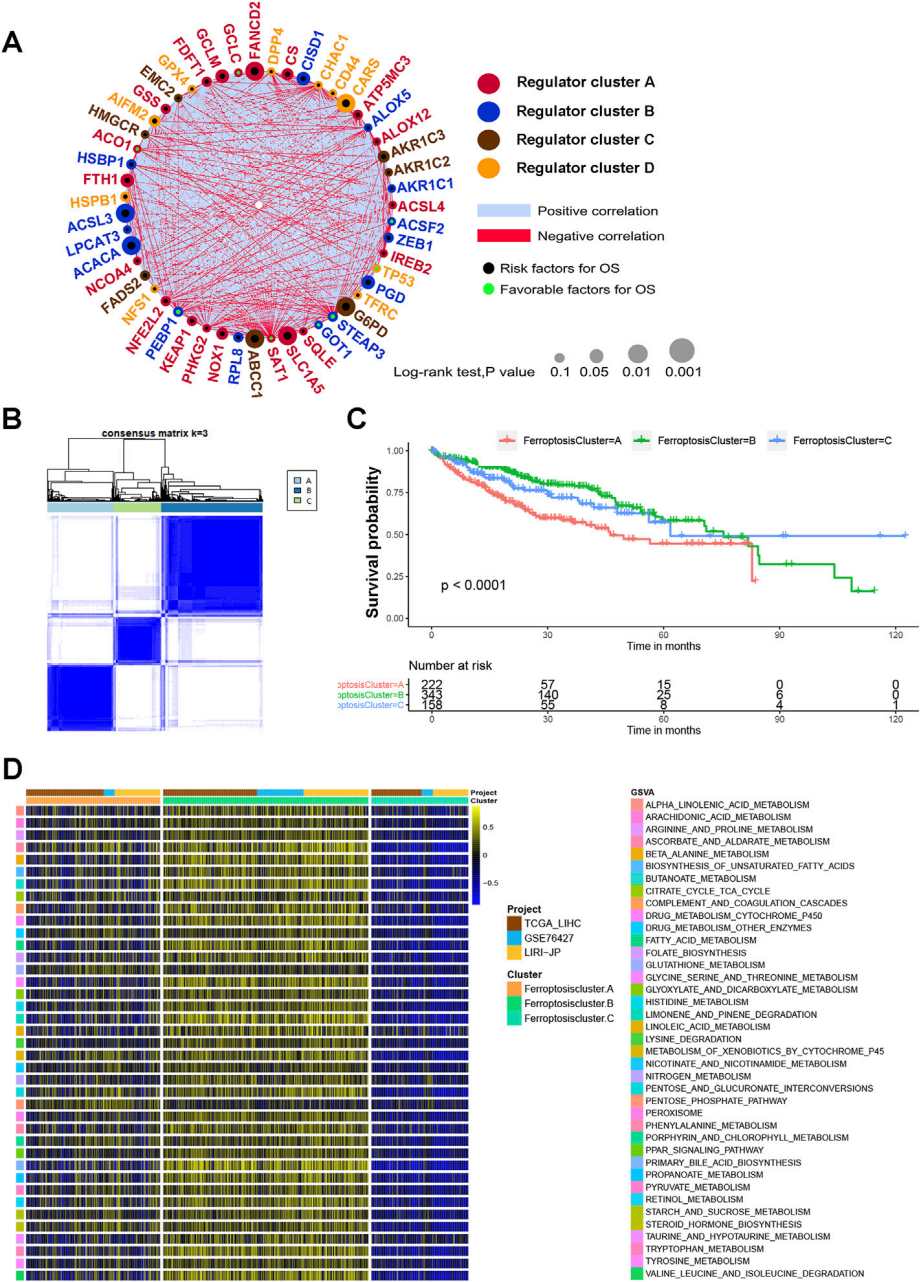
Ferroptosis modification patterns and relevant biological pathway. **(A)** The interaction of expression on 51 ferroptosis regulators in HCC. The circle size indicated the effect of each regulator on the prognosis. Black dots, risk factors for prognosis; Green dots, favorable factors for prognosis. Negative correlation was marked with red and positive correlation with blue. The regulator cell cluster **(A–D)** was marked with red, blue, brown and yellow, respectively. **(B)** Consensus matrices of 51 ferroptosis regulators in HCC for k = 3. **(C)** Survival analysis for the three ferroptosis modification subtypes for 723 HCC patients, including 222 cases in pattern A, 343 cases in pattern B and 158 cases in pattern C. **(D)** GSVA enrichment analysis showing the activation states of biological pathways in three distinct ferroptosis modification subtypes. The cohort composition TCGA-LIHC, GSE76427, LIRI-JP were used as sample annotations.

We next explored the interactions and prognostic significance between ferroptosis regulators and HCC patients using consensus clustering analysis. The comprehensive landscape of the interactions of the 51 ferroptosis regulators, the regulator connections and their prognostic significance in HCC patients was illustrated in the ferroptosis regulator network (Figure 2A and Supplementary Table S3). The results showed that the cross talking among ferroptosis regulators may play crucial parts in the formation of different ferroptosis modification patterns, and the ferroptosis regulator network was implicated in regulating tumorigenesis and progression of HCC. Next, the R package of ConsensusClusterPlus was used to classify HCC samples with qualitatively different ferroptosis modification patterns based on the expression of 51 ferroptosis regulators. Based on these hypotheses, we divided HCC patients into three distinct modification patterns via unsupervised clustering, including 222 cases in pattern A, 343 cases in pattern B and 158 cases in pattern C (Figure 2B and Supplemenraty Figure S2A,B). We termed these three patterns as ferroptosis cluster-A, ferroptosis cluster-B and ferroptosis cluster-C (Figure 2B). Survival analysis for the three ferroptosis modification subtypes showed the prominent survival advantage in ferroptosis-B modification subtype, whereas ferroptosis-A presented the worst prognosis (*p* < 0.0001) (Figure 2C). In addition, we utilized GSVA enrichment analysis to evaluate the biological molecular variations underlying three distinct ferroptosis modification subtypes (Supplemenraty Table S4). We were delighted to find that these three ferroptosis modification subgroups were enriched in different metabolic pathways respectively. As shown in Figure 2D, ferroptosis cluster-A was markedly enriched in glucose oxygenolysis pathways for example pentose phosphate pathway. Ferroptosis cluster-B presented amino acid metabolism enrichment in glycine serine and threonine metabolism, while ferroptosis cluster-C prominently related to steroid hormone biosynthesis pathway (Figure 2D).

### Correlation Between TME Infiltration Cells and Distinct Ferroptosis Modification Patterns

**FIGURE 3:**
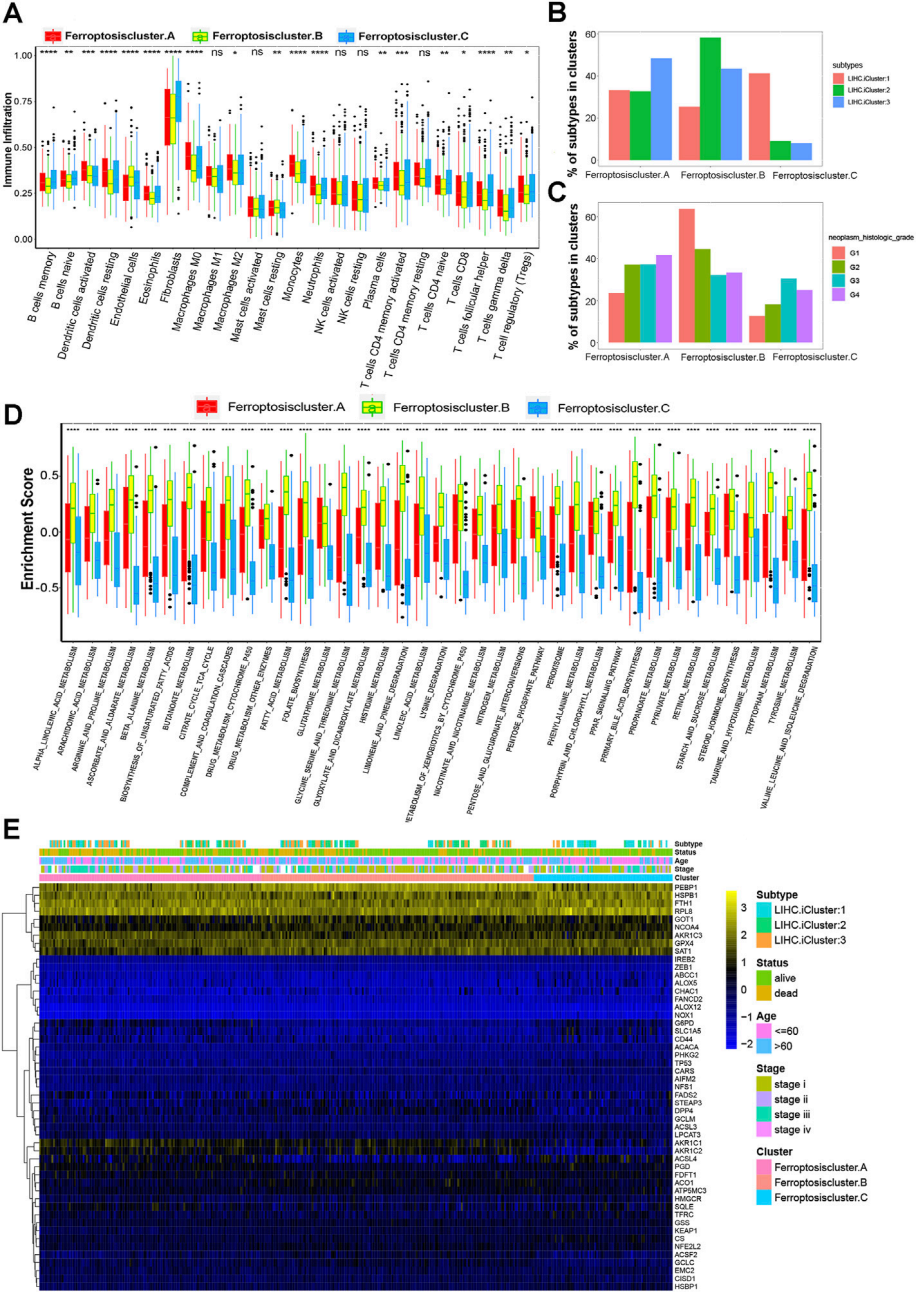
TME cell infiltration characteristics in distinct ferroptosis modification patterns. **(A)** The abundances of 24 immune infiltrating cell subpopulations in three ferroptosis modification patterns. **(B,C)** Correlations between ferroptosis modification patterns and clinicopathologic characteristics of HCC patients. **(B)** LIHC iCluster; **(C)** histologic grade. **(D)** GSVA enrichment pathways in three different ferroptosis subgroups using variance analysis. **(E)** Unsupervised clustering of 51 ferroptosis regulators in three ferroptosis modification subgroups of HCC.

In order to visualize and compare the relative abundances of 24 immune infiltrating cell subpopulations among distinct ferroptosis modification patterns, we performed ssGSEA analysis using R package of GSEABase. We found that three distinct ferroptosis patterns had significant differences in 19 of 24 immune infiltrating cells. Subsequent analyses of tumor cell infiltration indicated ferroptosis cluster-A was significantly enrich in immune cells including innate immune cell infiltration such as macrophage, eosinophil, dendritic cell, monocytes, neutrophils, mast cell and plasma cells (Figure 3A). Both tumor-promoting immune cells such as macrophages M2, and antitumor immune cells for example CD8 T cells and CD4 memory activated cells were also discovered enrich in ferroptosis cluster-A. It’s worth noting that patients in this ferroptosis modification pattern showed the worst prognosis (Figure 2C). We found that the majority of the immune cells were significantly reduced in ferroptosis cluster-B subtype (Figure 3A). Unexpectedly, patients in ferroptosis cluster B had the significant survival superiority compared with the other two clusters (Figure 2C). Ferroptosis cluster-C was enriched with fibroblasts, B memory cells, B naive cells and T follicular helper cells (Figure 3A). We then analyzed prognosis of 24 immune infiltrating cells in three distinct ferroptosis modification cluster by forest plot. The results revealed that B naive cells, mast resting cells, B memory cells, endothelial cells, CD4 T naive cells, dendritic activated cells, CD8 T cells, T gamma delta cells and CD4 T memory activated cells were protective factors for patients in HCC (Supplementary Figure S3A).

Next, we explored the correlations between ferroptosis modification patterns and clinicopathologic characteristics of HCC patients including patients’ race, gender, stage, liver cirrhosis, histological type, T stage and TCGA-LIHC molecular subtypes. We found that LIHC iCluster3, iCluster2 and iCluster1 had the highest proportion in ferroptosis cluster-A, ferroptosis cluster-B and ferroptosis cluster-C respectively (Figure 3B). The proportion of histologic grade G1 and G2, tumor stage I, pathology T1 and with no fibrosis were markedly high in ferroptosis cluster-B, while tumor stage III, pathology T4, histologic grade G4 and fibrous speta were significantly related with ferroptosis cluster-A, which might explain the very different prognosis between these two ferroptosis modification patterns (Figure 3C, Supplementary Fiugres S3B–E). The results demonstrated that distinct ferroptosis modification pattens might be used to evaluate certain clinical characteristics of patients (Figure 3B,C, Supplementary Fiugres S3B–F). Furthermore, we evaluated the enrichment scores of these three ferroptosis clusters on 39 GSVA enrichment pathways obtained from the previous analysis. The results showed that significant differences were found among three different ferroptosis subgroups in all 39 GSVA enrichment pathways by using variance analysis (Figure 3D). We further studied the expression differences of three ferroptosis modification subgroups in 51 ferroptosis regulator genes. As shown in Figure 3E and Supplementary Table S5, the heatmap indicated that ACLS4 in ferroptosis cluster-B was significantly downregulated than other two clusters, while AKR1C1 and AKR1C2 of ferroptosis cluster-C were significantly down expressed than other two clusters.

### Ferroptosis Phenotype Related DEGs in HCC

**FIGURE 4:**
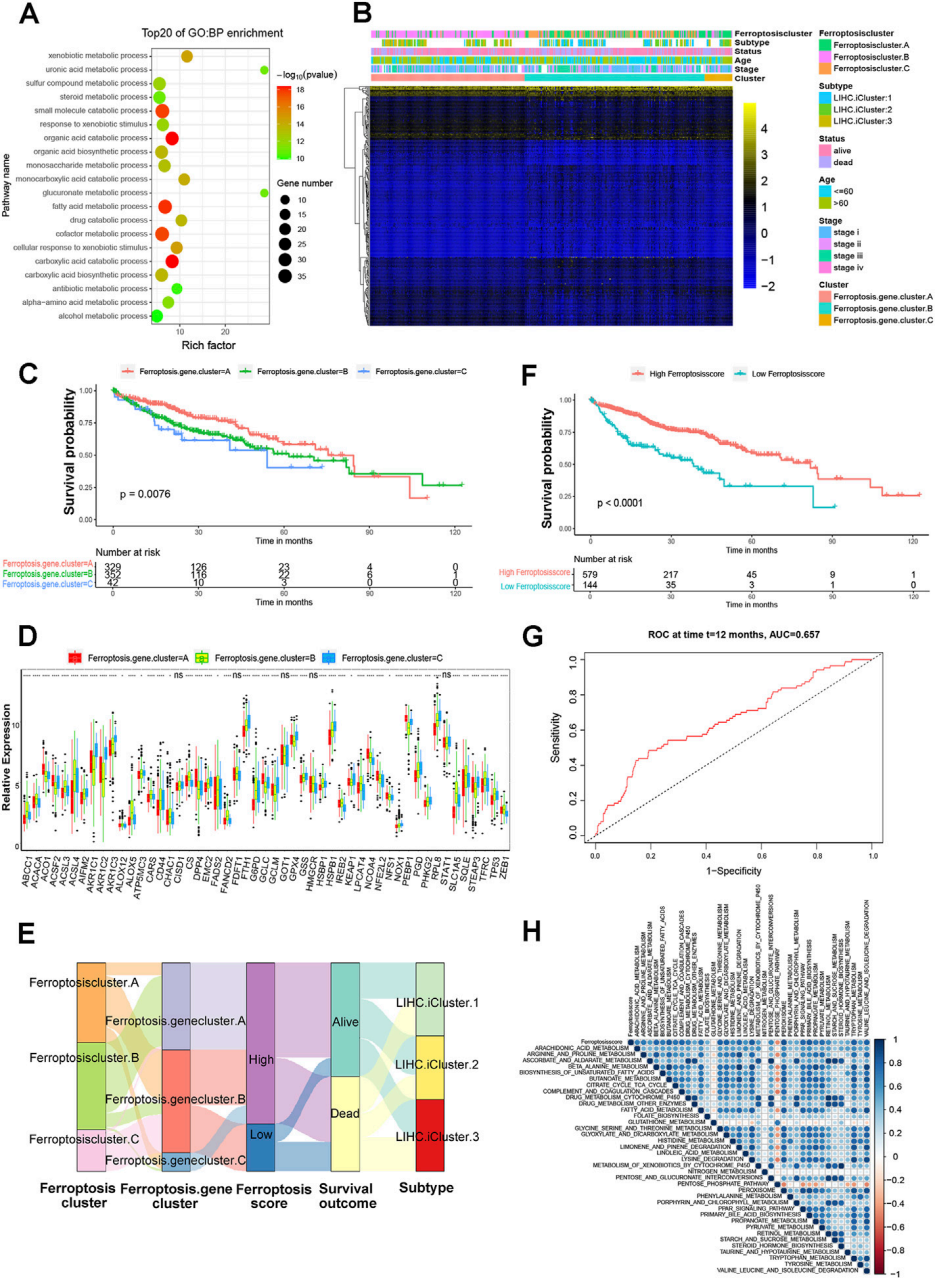
Construction of ferroptosis signatures. **(A)** Functional annotation for ferroptosis related genes using GO enrichment analysis. **(B)** Unsupervised clustering of overlapping ferroptosis phenotype-related DEGs to classify patients into different genomic subtypes, termed as ferroptosis gene cluster-A, ferroptosis gene cluster-B and ferroptosis gene cluster-C, respectively. The gene signature subtypes, ferroptosis clusters, molecular subtypes, tumor stage, status, and age were used as patient annotations. **(C)** The survival curves of the ferroptosis phenotype-related gene signatures were indicated by the Kaplan-Meier curves (*p* = 0.0076, Log-rank test). **(D)** The expression of 51 ferroptosis regulators in three gene cluster. The asterisks represented the statistical *p* value (*p < 0.05; **p < 0.01; ***p < 0.001). **(E)** Alluvial diagram showing the changes of ferroptosis clusters, LIHC molecular subtypes, ferroptosis gene cluster and ferroptosis score. **(F)** Kaplan-Meier curves for high and low ferroptosis score groups in HCC patients. Log-rank test, *p* < 0.001. **(G)** ROC curves to verify the prediction advantage of ferroptosisSig score in HCC patients. AUC = 0.657. **(H)** Correlations between ferroptosisSig score and the known biological gene signatures using Spearman analysis. The negative correlation was marked with red and positive correlation with blue.

Although the consensus clustering analysis according to ferroptosis regulator expression classified patients with HCC into three ferroptosis modification clusters, the underlying genetic alterations and expression patterns of these three distinct phenotypes were not well studied. Based on this, we next investigated the potential ferroptosis related transcriptional expression changes among three ferroptosis modification patterns in HCC. The limma R package was applied to determine overlapping differentially expressed genes (DEGs) among the three ferroptosis modification patterns. A total of 254 DEGs that represented the critical distinguishing index of the three ferroptosis modification patterns were considered as ferroptosis-related signature. GO enrichment analysis for the DEGs revealed that the biological processes related to organic acid catabolic process and fatty acid metabolic process using the R package of clusterProfiler (Figure 4A and Supplementary Table S6). Molecular function, cellular component and KEGG with enrichment were shown in Supplementary Figure S4A–C. To investigate the further regulation mechanism, we carried out unsupervised clustering analyses according to the 254 representative ferroptosis phenotype-related signature genes and patients were classified into three different genomic subtypes (Supplementary Figure S5A). Similar to the clustering subtype of ferroptosis modification patterns, three distinct ferroptosis modification genomic phenotypes were obtained named as ferroptosis gene cluster-A, ferroptosis gene cluster-B and ferroptosis gene cluster-C. In addition, GSVA enrichment analysis showed that ferroptosis gene cluster-A was markedly enriched in propanoate metabolism pathway. Ferroptosis gene cluster-B presented enrichment pathway associated with arginine and proline metabolism pathway, while ferroptosis gene cluster-C prominently related to lysine degradation pathway (Supplementary Figure S5B). These results revealed that three distinct ferroptosis modification patterns did present in HCC. We found that patients in ferroptosis gene cluster-A and B patterns were enriched in LIHC iCluster two and LIHC iCluster three molecular subtypes and with age more than 60-year-old, while patients with LIHC iCluster one molecular subtype and under the age of 60 years old were mainly concentrated in the ferroptosis gene cluster-B subgroup (Figure 4B). Next, 254 genes were included in univariate regression analysis, and 175 significant ones were identified as core genes (Supplementary Table S7). Subsequently, we performed random forest and principal component analysis, 91. genes were selected as ferroptosis gene signature. Survival analysis indicated that significantly difference among there ferroptosis gene clusters and ferroptosis gene cluster-A has the best progress (*p* = 0.0076, Figure 4C). The expression levels of the 51 ferroptosis regulators among three gene signature subgroups were also compared and shown in Figure 4D. We found that significant differences in 46 out of 51 ferroptosis regulator expression between the three ferroptosis gene signature subgroups, which was in accordance with the expected results of the ferroptosis modification patterns.

### Construction of the FerroptosisSig Score and Exploration of its Clinical Relevance

**FIGURE 5:**
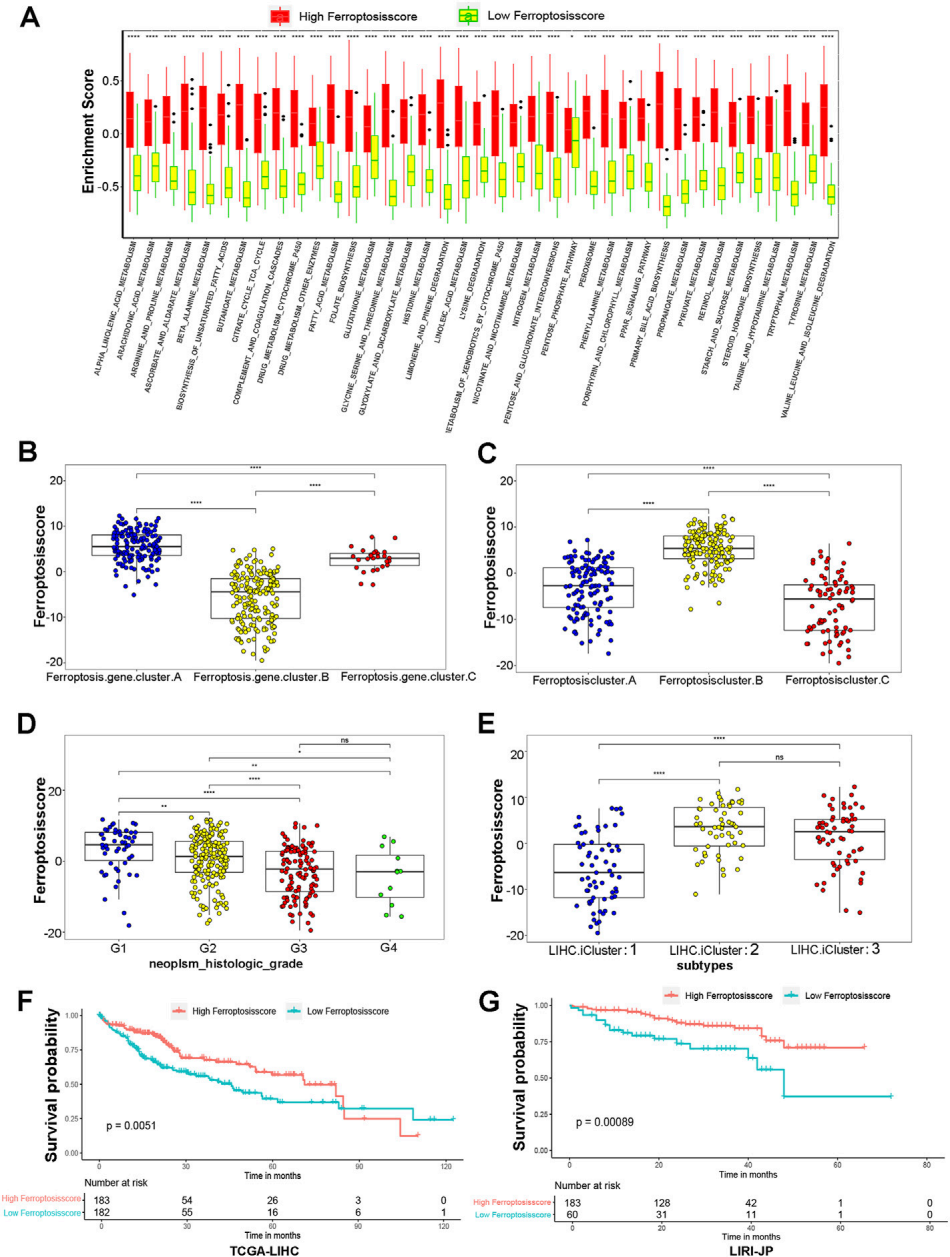
Characteristics of ferroptosisSig score and exploration of its clinical features. **(A)** GSVA enrichment pathways in two ferroptosisSig score subgroups using variance analysis. **(B–E)** Distribution of ferroptosisscore in distinct groups: **(B)** ferroptosis gene clusters, **(C)** ferroptosis clusters, **(D)** tumor histologic grade, **(E)** TCGA-LIHC molecular subtypes by Wilcox test. The asterisks represented the statistical *p* value (*p < 0.05; **p < 0.01; ***p < 0.001). **(F–G)** Kaplan-Meier curves for high and low ferroptosisSig score groups in HCC patients. **(F)** TCGA-LIHC cohort, Log-rank test, *p* = 0.0051. **(G)** LIRI-JP cohort, Log-rank test, *p* = 0.00089.

Considering the individual heterogeneity and complexity of ferroptosis modification, we developed a scoring scheme termed the ferroptosisSig score to quantify the ferroptosis modification pattern of individual HCC patients, which is based on the identified 91 ferroptosis-related signature genes. The alluvial diagram was used to visualize the attribute changes of individual patients (Figure 4E). Our results demonstrated that ferroptosis gene cluster-A with the LIHC iCluster1 subtype was linked to a higher ferroptosisSig score, whereas ferroptosis gene cluster-B exhibited a lower ferroptosisSig score. Furthermore, we divided the patients into low score or high score groups (Supplementary Figure S6A) and investigated the prognosis of the ferroptosisSig score in predicting clinical outcomes. The outcomes of high ferroptosisSig score patients were significantly better than those of low ferroptosisSig score group in HCC patients (*p* < .0001, Figure 4F). The results of the ROC curves analysis verified the prediction advantage of the established risk model (AUC = 0.657, Figure 4G). We evaluated the relationship between signaling pathways and the ferroptosisSig score using Pearson analysis. A heatmap of the correlation matrix indicated that the ferroptosisSig score was significantly positively associated with pentose phosphate pathway enrichment score but negatively correlated with butanoate metabolism pathway enrichment score (Figure 4H). Next, we investigated the enrichment scores of two ferroptosisSig subgroups on 39 GSVA enrichment pathways obtained from the previous analysis. The results revealed that significant differences were all found between low- and high-ferroptosisSig subgroups in all 39 GSVA enrichment pathways by wilcox test analysis (Figure 5A). Wilcox test revealed significant difference on ferroptosisscore between ferroptosis gene clusters. Ferroptosis gene cluster A showed the significantly increased score compared to the other two gene clusters and ferroptosis gene cluster B presented the lowest median score (Figure 5B). Moreover, ferroptosis cluster C showed the lowest median score while ferroptosis cluster B had the highest median score by wilcox test analysis (Figure 5C). We also explored the correlations between ferroptosisSig score and clinicopathologic characteristics of HCC patients. We found that patients’ race, gender, stage, histological grade, T stage and TCGA-LIHC molecular subtypes were significantly associated with ferroptosisSig score (Figures 5D,E, Supplementary Figures S6B–E). To further test the stability of ferroptosisSig score model, we validated the prognosis value of the ferroptosisSig score in clinical outcomes of three HCC database respectively, including TCGA-LIHC, GSE76427 and LIRI-JP cohorts (Supplementary Table S8). It showed that ferroptosisSig score displayed the potential prognosis predictive value in TCGA-LIHC cohort (*p* = .0051, Figure 5F) and LIRI-JP cohort (*p* = .00089, Figure 5G), and patients with high ferroptosisSig score indicated a prominent survival benefit. However, the survival outcome was opposite in GSE76427 cohort probably because of the less sample size (Supplementary Figure S6F). In addition, we performed univariate and multivariate cox regression analysis and the results demonstrated that ferroptosisSig score could be used as an independent predictor of prognosis (*p* < .05) (Supplementary Figure S6G,H). These results demonstrated that ferroptosisSig score may have potentially high value to evaluate certain clinical characteristics of patients and have better clinical benefit in HCC.

### The Role of FerroptosisSig Score in Predicting Immunotherapeutic Benefits

**FIGURE 6:**
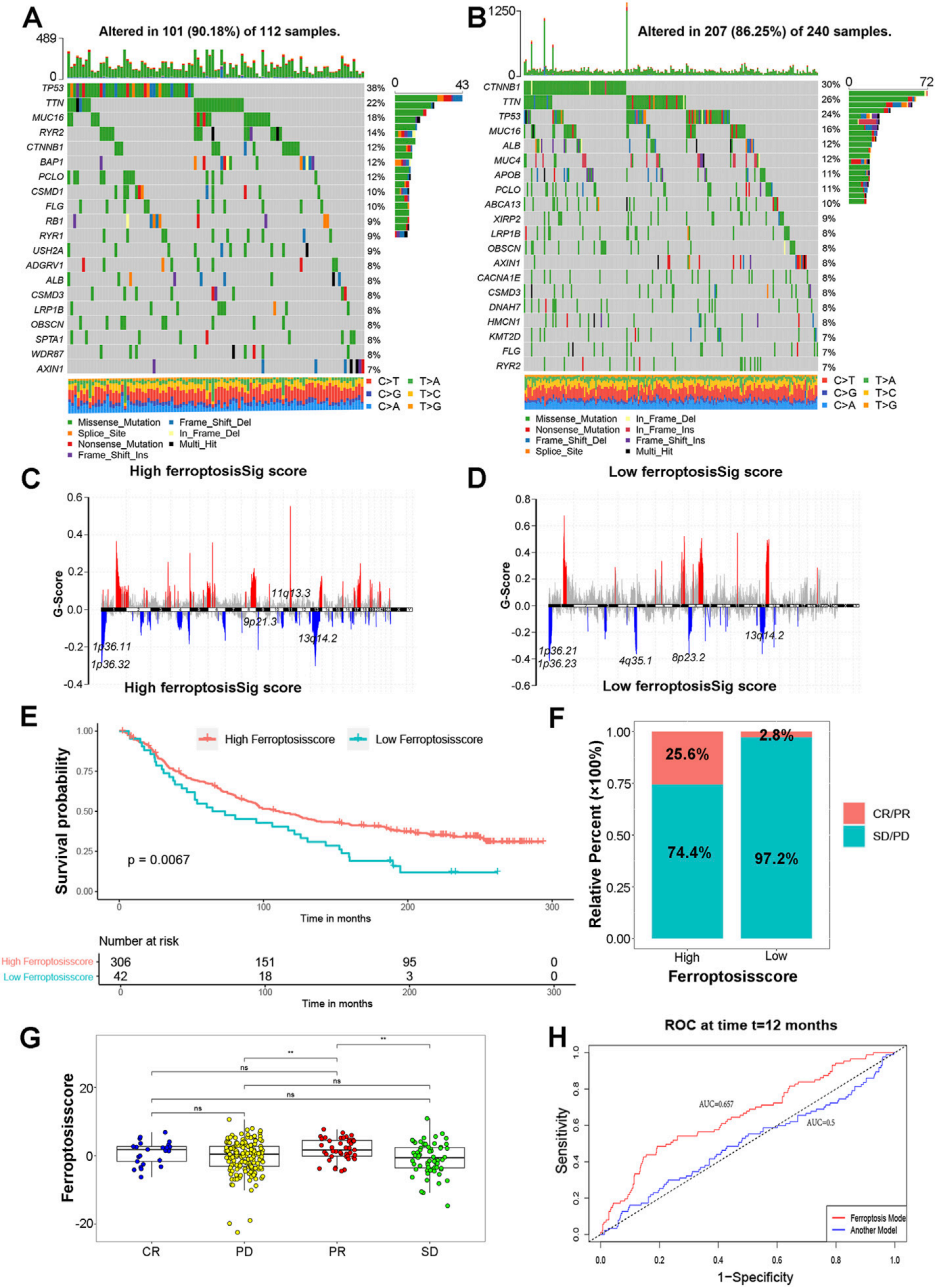
The role of ferroptosisSig score in predicting immunotherapeutic benefits. **(A,B)** The waterfall plot of tumor somatic mutation established by those with **(A)** high ferrptosisSig score and **(B)** low ferroptosisSig score. **(C,D)** CNV mutation G-score of **(C)** high ferrptosisSig score and **(D)** low ferroptosisSig score subgroup. **(E)** Kaplan-Meier curves for patients with high and low ferroptosisSig score subgroups in HCC patients. Log-rank test, *p* = 0.0067. **(F)** The proportion of patients with response to PD-1 blockade immunotherapy in low or high ferroptosisSig score groups. **(G)** Differences in ferroptosisSig score among distinct checkpoint blockade therapy response groups. The asterisks represented the statistical *p* value (*p < 0.05; **p < 0.01; ***p < 0.001). **(H)** The predictive value of the quantification of ferroptosisSig score in patients treated with anti-PD-1/L1 immunotherapy (AUC = 0.657).

Growing evidence has indicated important relationships between the tumor genome somatic mutations and response to immunotherapy. We then evaluated the distribution differences of somatic mutation for HCC patients in high ferroptosisSig score subgroup versus the low ferroptosisSig score subgroup in TCGA-LIHC cohort by maftools R package. The mutational landscapes indicated that TP53 (38% *VS* 24%) had higher somatic mutation rates in the high ferroptosisSig score subtype (Figure 6A), whereas CTNNB1 (30% *VS* 12%) had higher somatic mutation rates in the low ferroptosisSig score subtype (Figure 6B). To gain further insights into the somatic mutation chromosomal location in HCC, we analyzed the CNV mutation G-score of high and low ferroptosisSig score subgroup using gisticChromPlot R package. The top five q value of CNV mutation chromosomal location of high and low ferroptosisSig score subgroups were shown in Figures 6C,D. Taken together, these results presented us to profiles the effect of ferroptosisSig score classification on genomic variation and implied the potential complicated interaction between individual somatic mutations and ferroptosis modifications.

Immunotherapy represented by PD-1, PD-L1 and CTLA4 has attracted great interest based on the immune regulation of cancer cells. We wondered whether the ferroptosisSig score system could predict immune response of checkpoint blockade therapy based on IMvigor210CoreBiologies immunotherapy cohort. The results showed patients with high ferroptosisSig score presented a markedly prolonged survival (*p* = .0067, Figure 6E). We next explored the therapeutic evaluation of IMvigor210CoreBiologies cohort. It showed that high ferroptosisSig score displayed the significantly clinical benefits (*p* = 0.004455, Figure 6F). As shown in Figure 6G, we found that patients with high ferroptosisSig score showed a great survival advantage in partial response (PR) group compared with progression disease (PD) and stable disease (SD) groups (Figure 6G). To further verify the predictive effect of anti-PD/L1 immunotherapy by ferroptosisSig score, we performed ROC curves analysis in TCGA-LIHC cohort using a publicly published HCC prognostic model []. The risk score was calculated as follows: Risk score = 0.66 * (expression value of RNF24)+(−0.61) * (expression value of COPS8) + 0.40 * (expression value of EWSR1) +(−0.40) * (expression value of SUGCT) + (0.38) * (expression value of PCSK5) + (0.35) *(expression value of POLR3C)+ (0.31) * (expression value of NRBP1) +(0.27) * (expression value of MNAT1) +(0.18) * (expression value of EIF5B) +(−0.15) * (expression value of DUSP10) +(0.08) * (expression value of WASF1) +(0.07) * (expression value of CCDC88A)**.** We found that the AUC of the ferroptosisscore was 0.657 at 1 year significantly better than the published risk model, of which AUC was 0.5 (Figure 6H). Taken together, our study strongly indicated that ferroptosis modification signature might contribute to predicting the response to anti-PD-1/L1 immunotherapy in HCC.

Chemotherapy is one of the traditional therapies for HCC. In order to investigate the response of chemotherapy treatment, we next evaluated IC50 of Cisplatin and Gemcitabine in high and low ferroptosisSig score subgroups using pRRophetic R package. We found significant differences of IC50 between high and low ferroptosisSig groups in Cisplatin and Gemcitabine treatment groups, respectively (Supplementary Figure S6I,J). These results implied that ferroptosisSig score classification might also have potential predictive value on chemotherapeutic efficacy of HCC.

## Discussion

Ferroptosis is a newly discovered form of regulated cell death characterized by the accumulation of iron-dependent of lipid ROS in cells (Stockwell et al., 2017). It is different from other types of cell death such as apoptosis, necrosis, and autophagy (Elgendy et al., 2020). Recently, it has been explored that ferroptosis activation is one approach gaining a lot of attention to battle cancer (Elgendy et al., 2020). Immune infiltrating cells in the tumor microenvironment (TME) have been shown to play a key role in tumor progression and influence clinical outcomes in cancer patients. Mounting evidences emerged that ferroptosis took on a crucial role in synergistic effect on anti-tumor immunity (Hassannia et al., 2019; Mou et al., 2019). Wang et al. indicated that immunotherapy-activated CD8^+^ T cells induce ferroptosis in tumor cells *in vivo* and contribute to the anti-tumor efficacy of immunotherapy (Wang et al., 2019). As most previous studies mainly focused on single tumor immune infiltrating cell type or single ferroptosis regulator, the overall tumor microenvironment infiltration characterizations mediated by integrated ferroptosis regulators have not been comprehensively investigated. Therefore, unveiling distinct ferroptosis modification patterns in tumor immune microenvironment will highlight the connections of ferroptosis on anti-tumor immune response, and further offer more potential immunotherapy strategies for HCC patients.

The emergence of selective gene targeting therapy and immunotherapy has obviously improved the prognosis of cancer patients. The clinical treatment decisions are often based on abnormal molecular profiles or “signatures” rather than the tissue type or anatomical site of the tumor (Liu et al., 2020). For example, lung cancer bearing EGFR mutation, and colon cancer bearing KRAS mutation all benefit from inhibitors targeting mutation gene. Studies have shown that advanced HCC patients could benefit from multikinase inhibitor sorafenib targeted therapy (Llovet et al., 2008). In recent years, tumor immunotherapy such as anti-PD-1/PD-L1/CTLA-4 monoclonal antibody and chimeric antigen receptor T-cell (CAR-T) immunotherapy has extensively attentioned as an important part of combined therapy (Hilmi et al., 2019). Instead of targeting cancer cells directly, immunotherapy recruits and activates core immune guardian T cells to recognize and eliminate cancer cells through antigen antibody response (Muro et al., 2016). Considering that sorafenib and immunotherapy inhibit the growth of HCC via the induction of ferroptosis, HCC patients with different ferroptosis modification patterns and distinct immune infiltration cells might benefit differently from these treatments. In the current study, we determined three distinct ferroptosis modification patterns characterized by 51 ferroptosis regulatory genes. Ferroptosis cluster-A showed the presence of plenty of immune cells, while ferroptosis B had relatively few immune cells. Subsequent analysis indicated that patients in ferroptosis cluster B showed a survival advantage, while patients in ferroptosis modification A had the worst prognosis. Further studies showed low pathological grade and stage, early tumor stage and no fibrosis were significantly associated with ferroptosis cluster-B, while high pathological grade and stage, advanced tumor stage and fibrous speta were significantly related with ferroptosis cluster-A. Therefore, after fully exploring the clinicopathologic characterization by distinct ferroptosis modification patterns, it was not surprising that cluster A had the majority of immune cells but poorer prognosis and cluster B had fewest immune cells but improved prognosis.

Among the different cell death modalities, ferroptosis is unique in that it is mostly associated with several metabolic pathways, such as amino acid metabolism, fatty acid metabolism, (seleno)thiol metabolism, mevalonate pathway and mitochondrial respiration pathway (Stockwell et al., 2017). In this study, the ferroptosis cluster-A was markedly enriched in pentose phosphate pathway (PPP). Previous investigations showed that PPP can generate NADPH, which could positively regulate ferroptosis reckoned as a biomarker of ferroptosis sensitivity in many cancers (Zheng and Conrad, 2020). The PPP is required for fatty acid and plasma membrane synthesis in newly activated CD8^+^ T cells (Kidani et al., 2013). The ferroptosis cluster-B presented amino acid metabolism enrichment associated with glycine serine and threonine metabolism, which is very important for tumor immunity (Kelly and Pearce, 2020). The ferroptosis cluster-C prominently related to steroid hormone biosynthesis pathway, which could regulate CD8^+^ T Cell differentiation and in the tumor microenvironment (Acharya et al., 2020). Furthermore, our data also revealed a markedly correlation between ferroptosisSig score and metabolic pathway. Consistent with our previous results, PPP pathway which enzymes glucose-6-phosphate dehydrogenase and phosphogluconate dehydrogenase positively regulate ferroptosis (Gao et al., 2015).

Further study proved that differentially expressed genes (DEGs) between distinct ferroptosis modification patterns were significantly associated with ferroptosis and related biological pathways, suggesting that these DEGs were considered as ferroptosis phenotype-related gene signatures. Similar with the results of ferroptosis modification clusters, three transcriptomic subtypes based on ferroptosis signature genes were identified and were significantly related with different survival outcomes. Considering the individual heterogeneity of ferroptosis modification, it is urgent to quantify the ferroptosis modification patterns of individual tumor. Therefore, we developed a scoring system named “the ferroptosis gene signature” to evaluate the ferroptosis modification pattern of individual patients with hepatocellular carcinoma. The prognosis of high ferroptosisSig score patients were significantly better than patients with low ferroptosisSig score in HCC patients. What’s more, the ferroptosisSig score system was well validated to predict immune response of checkpoint blockade therapy by IMvigor210CoreBiologies immunotherapy cohort. A publicly published TCGA-LIHC cohort with anti-PD/L1 immunotherapy prognostic model was further well verified the predictive effect of ferroptosisSig score. Considered together, these data suggest that our hypothesis that the ferroptosis modification pattern could be applied in clinical practice to guide therapeutic regimens.

Evaluation of mutation driver genes in human tumors is a critical basis for cancer diagnosis, treatment and rational therapy selection. In particular, we observed that patients in the high ferroptosisSig score subtype had a significantly higher mutation frequency of TP53 (38% *VS* 24%), whereas CTNNB1 (30% *VS* 12%) had higher somatic mutation rates in the low ferroptosisSig score subtype. TP53 is considered to be a highly specific marker of malignant tumors and frequently mutated in many tumor types, including HCC (Khemlina et al., 2017; Long et al., 2019). Previous studies showed that TP53 mutation could induce or inhibit ferroptosis depending on the variation mutations of genes. It is supposed that more studies are needed to elucidate the exact effects on ferroptosis of HCC. Furthermore, many studies reported that TP53 mutations could suppress antitumor immune response via regulation of PD-L1 or NK cell ligands, while a few studies showed that TP53 mutations could promote antitumor immunity (Jiang et al., 2015; Tarangelo et al., 2018). CTNNB1 which is involved in the beta-catenin signaling pathway usually inhibits immunity response of tumor microenvironment (Thorsson et al., 2018; Ruiz de Galarreta et al., 2019). Consequently, these ferroptosisSig score related tumor driver gene mutations might contribute to the complicated interaction of immune activity and ferroptosis modification.

## Conclusion

In conclusion, we systematically evaluated regulation mechanisms of ferroptosis modification patterns with tumor immune microenvironment infiltration characteristics. The variation of ferroptosis modification patterns was an important factor leading to the heterogeneity and complexity of individual tumor immune microenvironment. These ferroptosis modification patterns could be considered to guide the clinic immunotherapy strategies.

## Data Availability

All data used in this work can be acquired from the Gene-Expression Omnibus (GEO; https://www.ncbi.nlm.nih.gov/geo/) under the accession numberS GSE76427 and GSE14520, Cancer Genome Atlas (TCGA) database (https://cancergenome.nih.gov/), International Cancer Genome Consortium (ICGC) database (https://icgc.org/) and the GDC portal (https://portal.gdc.cancer.gov/).

## Abbreviations

AUC, area under the curve; CAR-T, antigen receptor T-cell; CNV, Copy number variation; DEGs, differentially expressed genes; DOFs, drivers of ferroptosis; FRGs, ferroptosis regulator genes; GDC, Genomic Data Commons; GEO, Gene Expression Omnibus database; GO, gene ontology; GSEA, gene set enrichment analysis; GSVA, gene set variation analysis; HCC, hepatocellular carcinoma; ICIs, immune checkpoint inhibitors; ICGC, International Cancer Genome Consortium; NADPH, nicotinamide adenine dinucleotide phosphate; PCA, principal component analysis; PD, progression disease; PPP, pentose phosphate pathway; PR, partial response; ROC, receiver operating characteristic; ROS, reactive oxygen species; SD, stable disease; SOFs, suppressors of ferroptosis; TCGA, Cancer Genome Atlas database; TME, tumor microenvironment.
