# Supplementary figures and images for: Ferroptosis Regulator Modification Patterns and Tumor Microenvironment Immune Infiltration Characterization in Hepatocellular Carcinoma
Source: Front Mol Biosci. 2022 Jan 28;9:807502. doi: 10.3389/fmolb.2022.807502 (PMC8832196; doi:10.3389/fmolb.2022.807502)

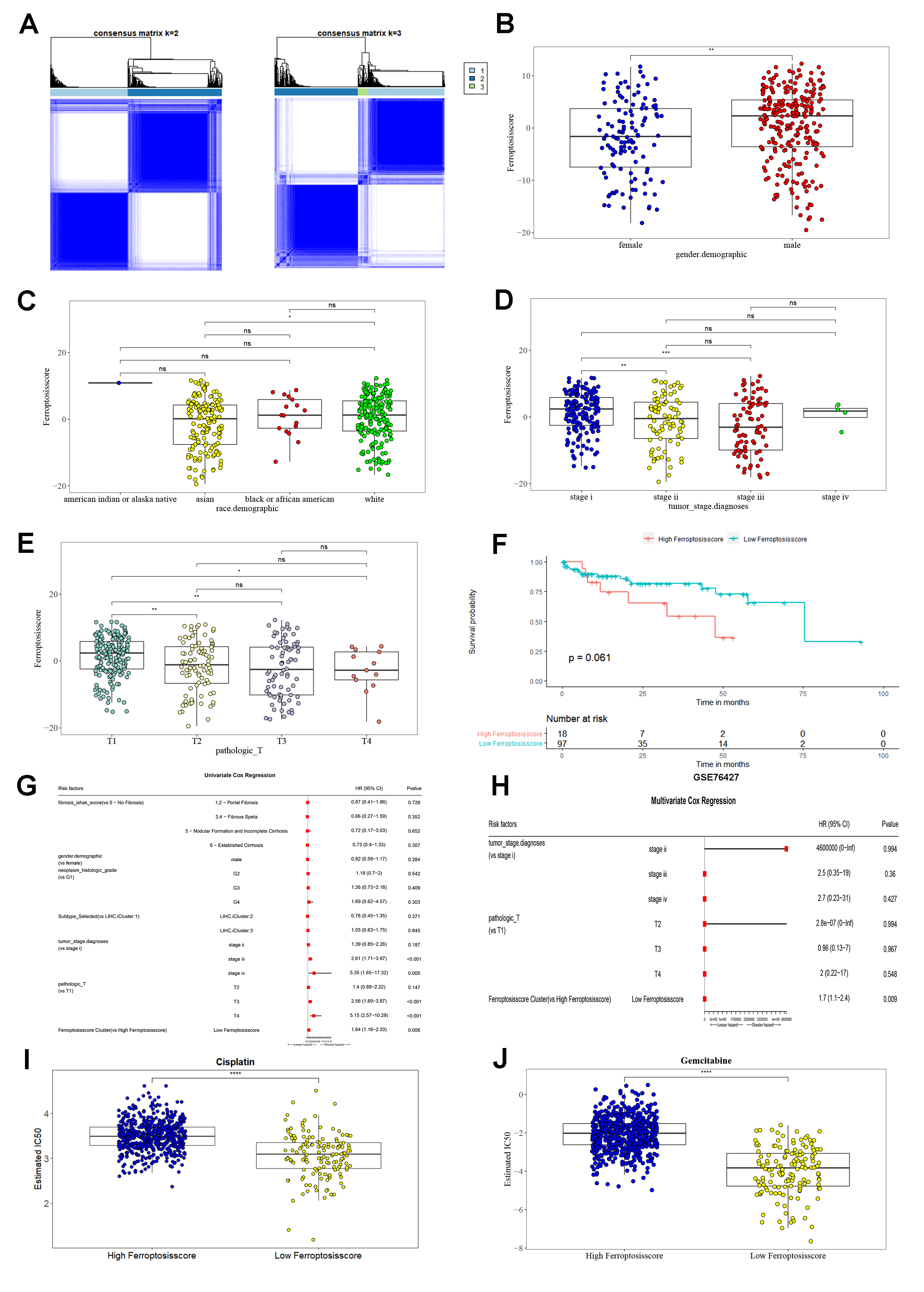

Supplement: Supplementary file 1 [file Image6.TIF]

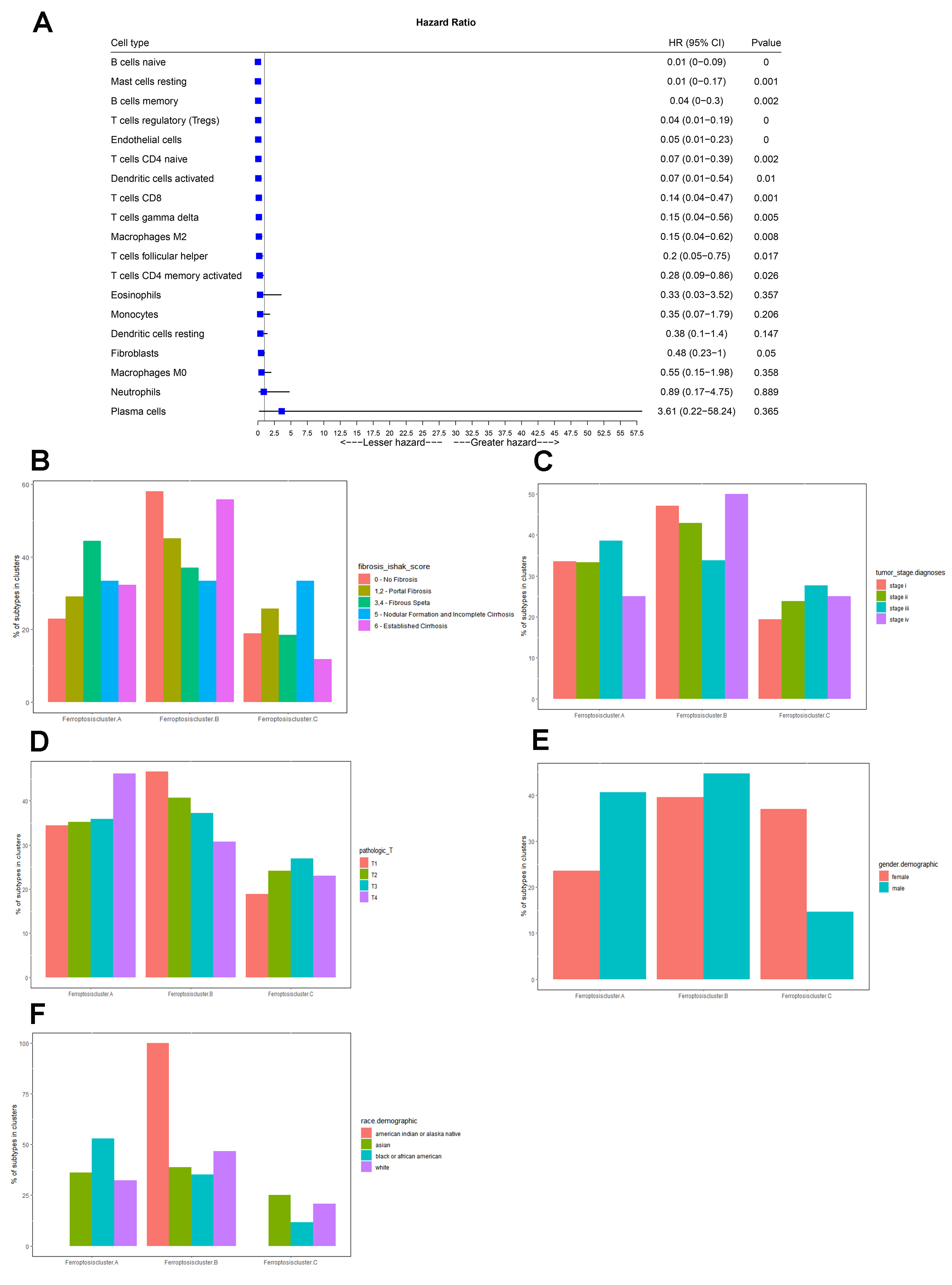

Supplement: Supplementary file 2 [file Image3.TIF]

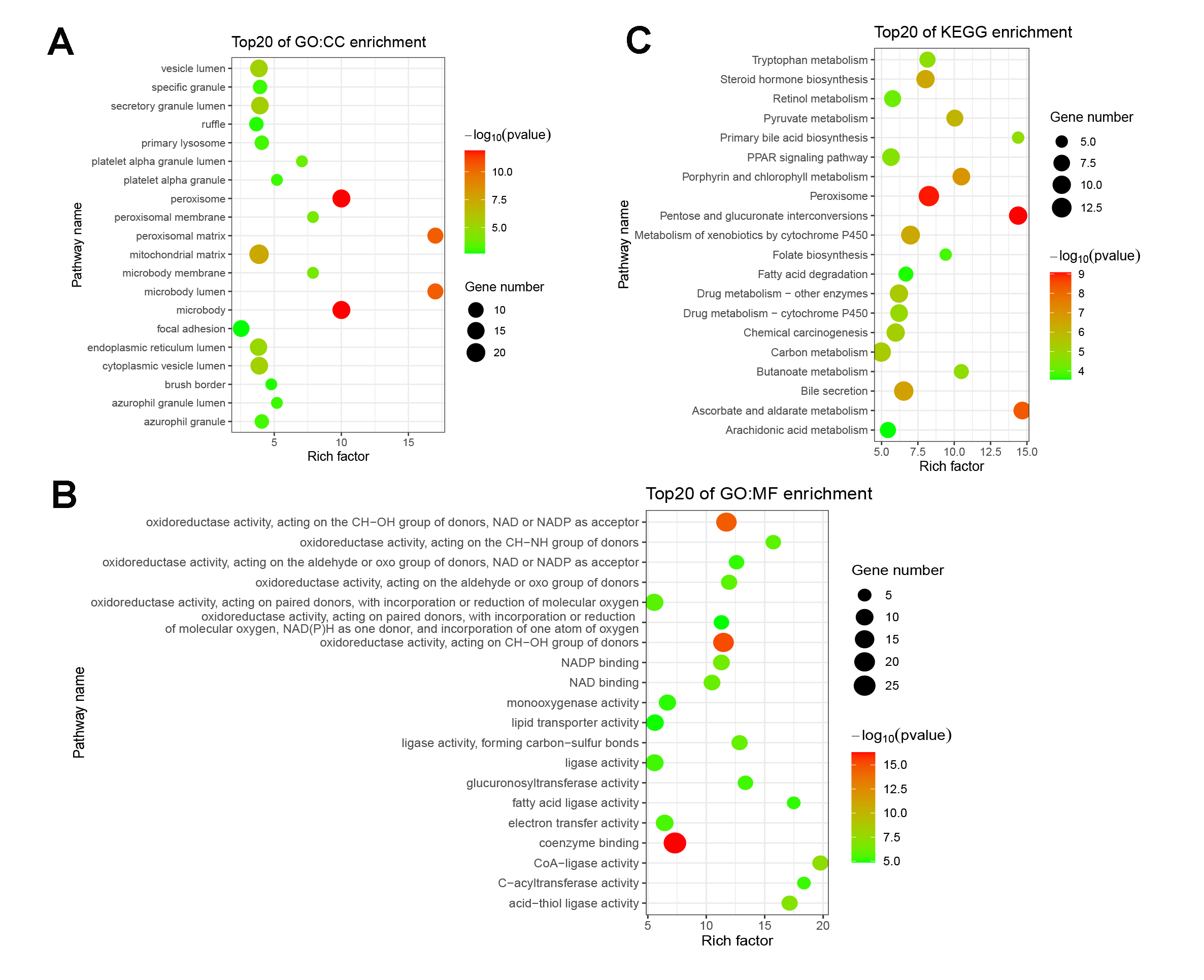

Supplement: Supplementary file 3 [file Image4.TIF]

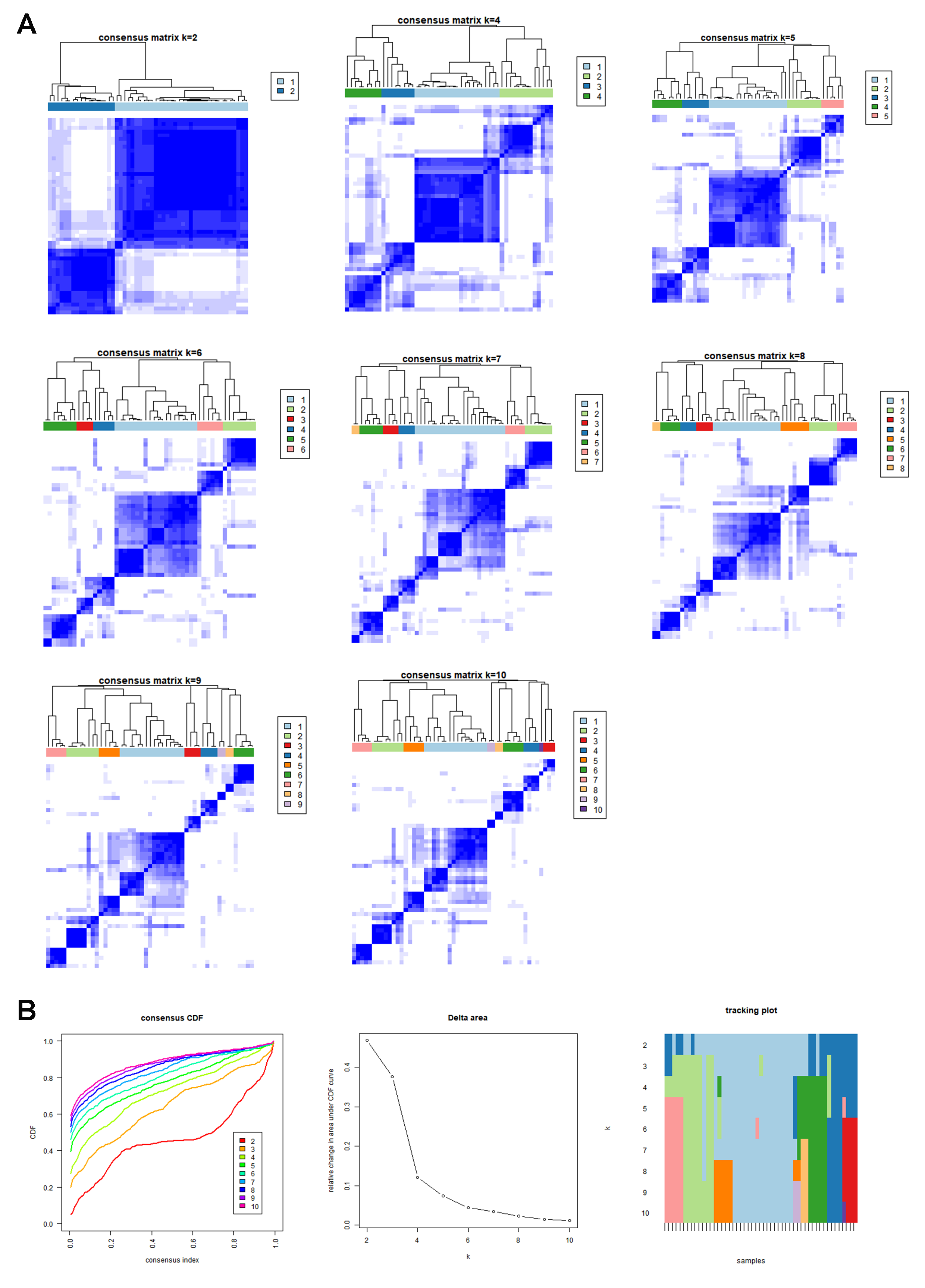

Supplement: Supplementary file 4 [file Image2.TIF]

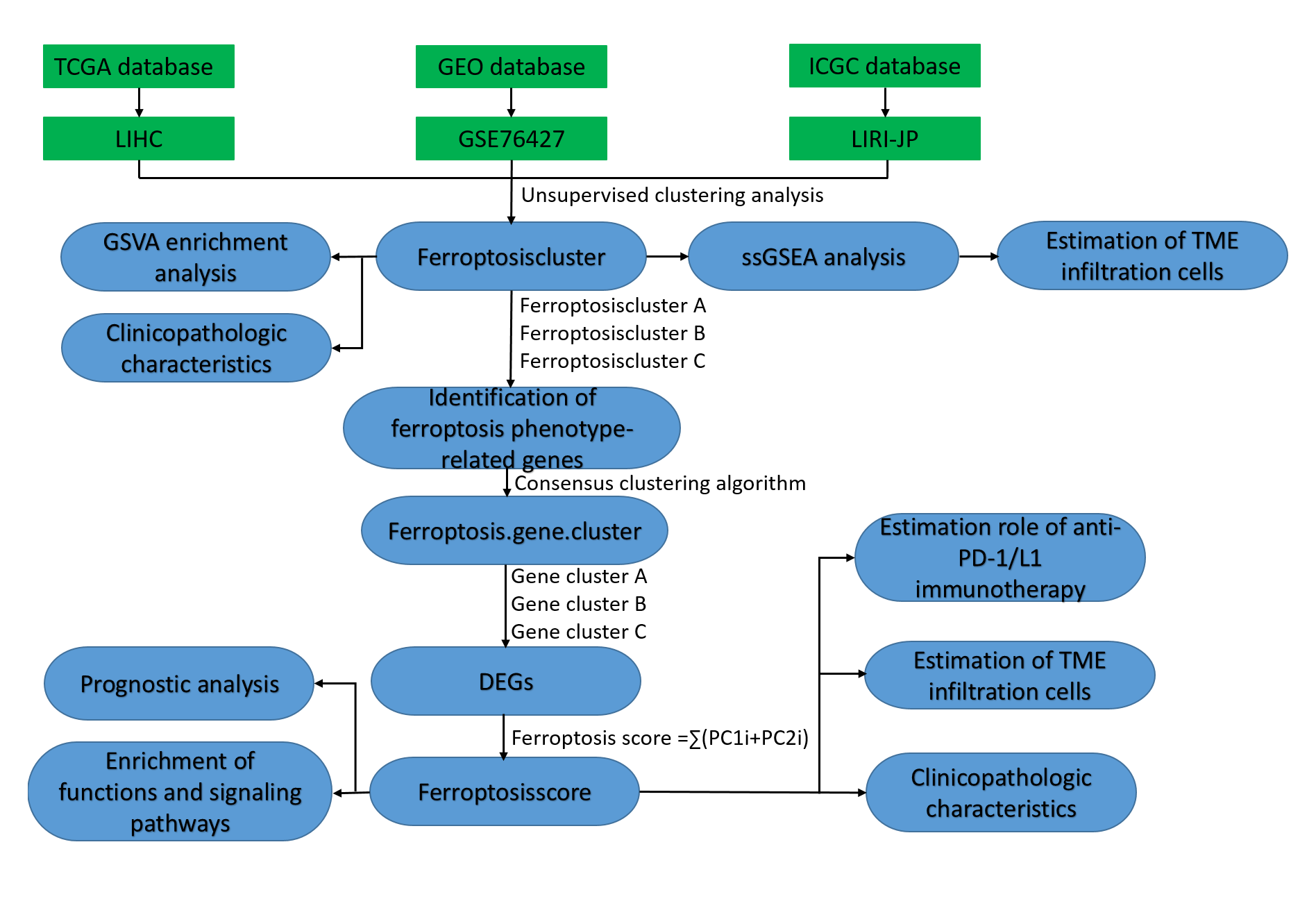

Supplement: Supplementary file 5 [file Image1.TIF]

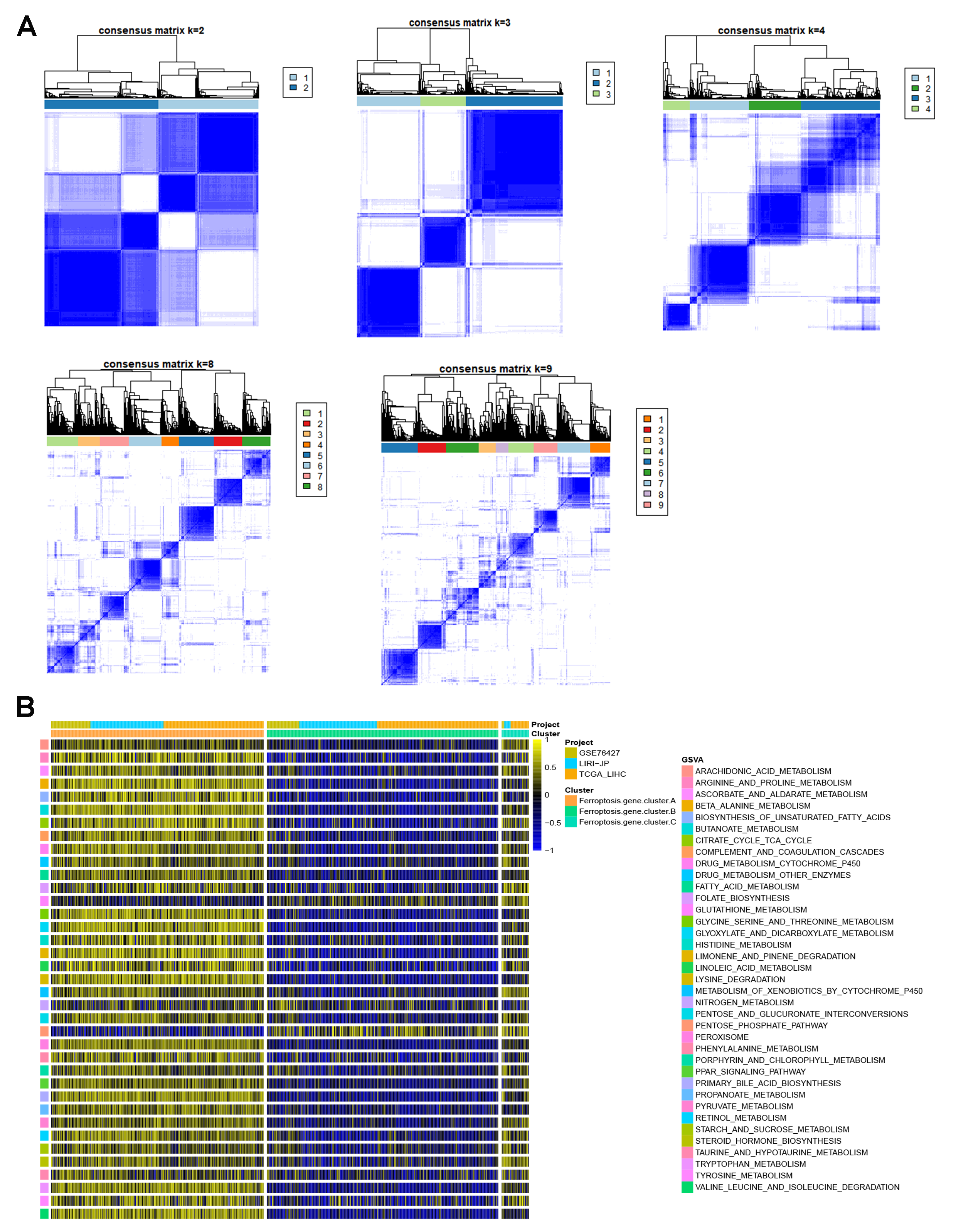

Supplement: Supplementary file 6 [file Image5.TIF]
